# Lactational Responses of Heat-Stressed Dairy Goats to Dietary L-Carnitine Supplementation

**DOI:** 10.3390/ani9080567

**Published:** 2019-08-16

**Authors:** Nabil Mehaba, Ahmed A. K. Salama, Xavier Such, Elena Albanell, Gerardo Caja

**Affiliations:** Ruminant Research Group (G2R), Animal and Food Science Department, Universitat Autònoma de Barcelona, Bellaterra, 08193 Barcelona, Spain

**Keywords:** L-carnitine, heat stress, feed sorting, metabolism, dairy goats

## Abstract

**Simple Summary:**

Endogenous carnitine synthesis is reduced by heat stress, and we hypothesized that carnitine supplementation would improve lipid metabolism and performance of dairy goats when exposed to high ambient temperature. In the current study, goats were exposed to thermal-neutral (15 to 20 °C) or heat stress (28 to 35 °C) conditions. In each environmental condition, goats were supplemented or not with rumen-protected carnitine in their diets. Goats exposed to heat stress experienced high body temperatures and respiratory rates, and reduced feed intake and milk production. Carnitine supplementation was transferred efficiently to blood, but had no effect on physiological or productive parameters in goats. We conclude that extra carnitine has no beneficial effects on goats’ performance and is not needed in thermal-neutral or heat stress conditions.

**Abstract:**

Heat stress causes significant losses in milk production, and nutritional strategies are needed to alleviate its effects. Endogenous carnitine synthesis is also reduced by heat stress (HS). Carnitine plays a central role in fatty acid oxidation and buffers the toxic effects of acyl groups. We hypothesized that carnitine supplementation would make up for any carnitine deficiencies during HS and improve lipid metabolism. The objective was to evaluate rumen-protected L-carnitine (CAR) supplementation in dairy goats under thermo-neutral (TN) or HS conditions. Four Murciano-Granadina dairy goats were used in a four × four Latin square design. Goats were allocated to one of four treatments in a two × two factorial arrangement. Factors were 1) diet: control (CON) or supplementation with CAR (1 g/d); and 2) ambient conditions: TN (15 to 20 °C) or HS (0900 to 2100 h at 35 °C, 2100 to 0900 h at 28 °C). Blood free-, acetyl-, and total-carnitine concentrations increased almost three times by supplementation. Despite this efficient absorption, CAR had no effect on feed intake, milk production or blood metabolites in TN or HS conditions. Heat stress increased rectal temperature and respiratory rate. Additionally, HS goats experienced 26% loss in feed intake, but they tended to eat longer particle sizes. Compared to TN, heat-stressed goats lost more subcutaneous fat (difference in fat thickness measured before and after each period = −0.72 vs. +0.64 mm). In conclusion, supplemented L-carnitine was efficiently absorbed, but it had no lactational effects on performance of goats under thermo-neutral or heat stress conditions.

## 1. Introduction

Heat stress (HS) causes significant losses in milk yield and milk components in dairy animals [1,2]. Goats are considered more tolerant to HS compared to dairy cows because of their greater sweating rate and lower body weight (BW): surface ratio, allowing greater heat dissipation [3]. However, lactating dairy goats exhibit several changes in performance due to HS, including reductions in feed intake, milk yield, milk fat, and milk protein [3,4].

The HS compromises animal performance by directly altering metabolism and the hierarchy of nutrient utilization [5]. For instance, heat-stressed (HSed) goats challenged with epinephrine have lower blood concentrations of non-esterified fatty acids (NEFA) compared to goats under thermal-neutral (TN) conditions, indicating that lipid tissue of HSed goats becomes less sensitive to lipolytic signals [3]. Additionally, blood NEFA and ß-hydroxybutyrate (BHB) do not vary between HS and TN goats [3] or cows [1], although they experience negative energy balance. These findings might indicate that HSed animals are not able to use body fat reserves to cover their energy needs. Recently, blood transcriptomics of HSed goats showed the downregulation of several anti-inflammatory pathways, indicating that HSed goats could be in an inflammation status [6]. Inflammation impairs peroxisome proliferator-activated receptor-regulated fatty acid oxidation and carnitine synthesis from lysine [7]. Thus, HSed animals may need more carnitine compared to TN animals.

L-carnitine (CAR) has two main roles in eukaryote cells: 1) A “shuttle role”, in the transfer of long-chain fatty acids from cytosol to mitochondria for subsequent β-oxidation and the production of acetyl-CoA used for energy production (by ketogenesis or in TCA cycle); and 2) the “buffer role” of the acyl groups (by modulation of the acyl-CoA/CoA and reduction of the acyl toxicity by excreting them as carnitine esters) [8].

Heat stress blocks fat mobilization in dairy cows [1,2] and apparently it does the same in dairy goats [3]. However, Hamzaoui et al. [4] exposed goats to HS for four weeks, and reported that blood NEFA levels are greater in HS in the first week than TN, but for the remaining weeks no differences in NEFA are detected. This finding might indicate that HSed goats start to mobilize lipid, but as they are not able to use these fatty acids (presumably because synthesis of carnitine is reduced as indicated above) they stop mobilizing lipid tissue despite the decreased energy intake. Randle [9] reported that the provision of free fatty acids promotes fatty acid oxidation and storage, inhibits glucose oxidation and may promote glucose storage if glycogen reserves are incomplete. Therefore, we hypothesized that CAR supplementation would help in the utilization of fatty acids and improve energy metabolism. The objective of the current study was to determine the influence of L-carnitine supplementation on the physiological and lactational responses in dairy goats under heat stress conditions. To date and to the best of our knowledge, no information is available on the effects L-carnitine supplementation in heat-stressed dairy goats.

## 2. Materials and Methods

Animal care conditions and management practices agreed with the procedures stated by the Ethical Committee of Animal and Human Experimentation of the Universitat Autònoma de Barcelona (CEEAH reference 11/1430) and the codes of recommendations for the welfare of livestock of the Ministry of Agriculture, Food and Environment of Spain.

### 2.1. Animals, Treatments, and Management Conditions

Four multiparous Murciano-Granadina dairy goats (134 ± 2 days in milk; 2.48 ± 0.02 L/d milk yield; 46.1 ± 0.5 kg BW) with healthy and symmetrical udders from the herd of the experimental farm of the Universitat Autònoma de Barcelona were used. The design was 4 × 4 Latin square with 16-d periods. The first 11 d were for adaptation, while the last 5 d were the measurement period. Dietary L-carnitine supplementation increases carnitine levels in blood, milk, liver, and muscles within 1 wk in dairy cows [10]. Consequently, we considered the 16-d experimental periods enough time to study the possible CAR effects.

Goats were allocated to one of 4 treatments in 2 × 2 factorial arrangements. Factors were: 1) Diet: control without supplementation (CON), or supplementation with 5 g of rumen-protected CAR (20% pure L-carnitine; CarnEon20 Rumin-Pro, Kaesler Animal Nutrition, Cuxhaven, Germany); and 2) ambient conditions: TN (15 to 20 °C and 40% to 70% relative humidity throughout the day) or HS (from 0900 to 2100h at 35 °C and from 2100 to 0900h at 28 °C with 45% ± 5% relative humidity). This resulted in 4 treatment combinations: TN-CON, TN-CAR, HS-CON, and HS-CAR.

The temperature humidity index (THI) values were calculated according to NRC [11]: THI = (1.8 × T_db_ + 32) − [(0.55 − 0.0055 × RH) × (1.8 × T_db_ − 26.8)], where T_db_ is the dry bulb temperature (°C) and RH is the relative humidity (%). The THI values for TN varied between 59 and 65 throughout the day, whereas for HS, the THI values were 84 and 75 for the day and night, respectively. These THI values used in the current experiment for HSed goats are classfied as “alert” (80 ≤ THI < 85) and “normal” (THI < 80) stress levels during the day and night, respectively [12]. The photoperiod was maintained constant 12–12 h (day, 0900 to 2100; night, 2100 to 0900) in both TN and HS conditions. Data of environmental temperature and humidity were recorded every 10 min by using 2 data loggers (Opus 10, Lufft, Fellbach, Germany).

Throughout the experiment, (mid-March to mid-June), the TN goats were kept indoors, and the temperature was maintained at 15 to 20 °C with the help of an electric heater equipped with a thermostat (3.5 kW; General Electric, Barcelona, Spain) when necessary. Temperature and relative humidity averaged 17.4 ± 0.5 °C and 62% ± 5% (THI = 63) for the TN goats. The HS goats were kept in a 4 × 6 × 2.3 m climatic chamber (Euroshield, ETS Lindgren-Euroshield Oy, Eura, Finland) provided with a temperature and humidity controlling system (CAREL Controls Ibérica, S.L., Barcelona, Spain). A continuous 90 m^3^/h air turnover was maintained throughout the experiment.

Goats had a 4-wk pre-experimental period under TN conditions for the adaptation to the diet and to the experimental conditions before applying the ambient conditions. When goats were switched from TN to HS conditions, the temperature increased in 2 steps (1 d at 25 °C and 1 d at 30 °C, 45 ± 5% humidity), but no transition was applied for the change from HS to TN.

Goats were milked twice daily (0800 and 1700 h) using a portable milking machine set at 42 kPa, 90 pulses/min, and 66% pulsation ratio, provided with recording jars (5 L ± 5%). Milking routine included cluster attachment without udder preparation or teat cleaning, machine milking, machine stripping before cluster removal, and teat dipping in an iodine solution (P3-ioshield, Ecolab Hispano-Portuguesa, Barcelona, Spain).

The 5 g of CAR supplement were daily weighed by an electronic scale (Mobba Barcelona, Barcelona, Spain), mixed with 50 g crushed barley, and offered individually before the morning milking. The CON goats were also fed 50 g crushed barley without CAR supplementation. The CAR product contained 11.6% crude proein and 50.8% ether extract on dry matter (DM) basis. The 5 g daily dose of the commercial CAR supplement provided 1 g pure L-carnitine. The CAR dose (1 g) in the current study is equivalent to 0.056 g/kg BW^0.75^, which is similar to the dosage used by LaCount et al. [13] in dairy cows (0.054 g/kg BW^0.75^), although LaCount et al. [13] used rumen-unprotected L-carnitine. We chose to use this similar dose despite the fact that our product is rumen-protected because our HSed goats presumably need more carnitine as mentioned in the introduction.

The daily total mixed ration was distributed individually to each goat once daily after the morning milking and adjusted at 30% leftover based on the previous day intake. The ration was formulated to cover requirements according to the Institut National de la Recherche Agronomique (INRA) [14] and consisted of (as fed) alfalfa hay 60.4%, ground barley grain 15%, beet pulp 9.1%, crashed corn grain 7.5%, soybean meal 3%, sunflower meal 3%, molasses 1%, salt 0.6%, sodium bicarbonate 0.2%, and vitamin–mineral corrector for goats 0.2%. The chemical composition and nutritive value of the ration are shown in Table 1. Mineral and vitamin blocks were freely available to each goat (Na, 36.74%; Ca, 0.32; Mg, 1.09%; Zn, 5 g/kg; Mn, 1.5 g/kg; S, 912 mg/kg; Fe, 304 mg/kg; I, 75 mg/kg; Co, 50 mg/kg; Se, 25 mg/kg; Ovi bloc, Sal Cupido, Barcelona, Spain). Clean water was permanently available at ambient temperature.

### 2.2. Sample Collection, Analyses, and Measurements

Rectal temperatures and respiratory rates were daily recorded at 0800, 1200, and 1700 h. Rectal temperature was measured by a digital clinical thermometer (Model ICO Technology “mini color”, Barcelona, Spain; range, 32.0 to 43.9 °C; accuracy, ± 0.1 °C), whereas number of inhalations and exhalations counted during 60 s indicated the respiratory rate.

Feed intake was recorded daily throughout the experiment. A feed sample was collected before the beginning of the experimental period and was ground through a 1 mm stainless steel screen, and then analyzed for DM, acid detergent fiber, neutral detergent fiber, and ash according to the official analytical methods [15]. The Dumas method [15] with a Leco analyzer (Leco Corporation, St. Joseph, MI) was used for crude protein determinations.

A sample of 5% total orts was collected daily during the measurement period and mixed together to make a composite of orts per each goat and period. Particle size of the ration and orts were measured according to Heinrichs and Kononoff [16]. The Penn State Particle Separator (DSE 2013-186, PA) was used. The average particle size was calculated using a spreadsheet downloaded from the Penn State Extention Website (https://extension.psu.edu/penn-state-particle-separator). 

Milk yield of individual goats was weighed at each milking throughout the experiment using an electronic scale (Mobba Barcelona). Milk composition was evaluated for 2 d (d 14 and 15 of each period). A composite milk sample from the morning and afternoon milkings (approximately 100 mL) was collected and preserved with an antimicrobial tablet (Bronopol, Broad Spectrum Microtabs II, D&F Control Systems, San Ramon, CA) at 4 °C until analysis. Refrigerated milk samples were sent to the Laboratori Interprofessional Lleter de Catalunya (ALLIC, Cabrils, Barcelona, Spain) for the analyses of major components (total solids, fat, protein, and lactose) using medium infrared spectrophotometry (MilkoScan FT2, Foss, DK-3400 Hillerød, Denmark), and somatic cell count using an automatic cell counter (Fossomatic 5000, Foss Electric, Hillerød, Denmark) previously calibrated for goat milk.

Goats were weighed before feeding at the beginning and the end of each experimental period by a scale (model Tru-Test AG500 Digital Indicator, Auckland, New Zealand; accuracy, ± 0.5 kg). The scale was calibrated by a 5 kg weight before every weighing. The subcutaneous fat thickness was measured by ultrasonography according to Teixeira et al. [17]. The ultrasound images were taken using a VET 180 Plus ultrasound (Sonosonite, Bothell, WA) with a 5 MHz probe (veterinary model). When the ultrasound images were taken at the breast bone, goats were restrained in dorsal recumbency on a table. The fat thickness was measured in a perpendicular position to the ventral midline at the level of the third and fourth sternebrae. Images were then processed using an image analysis program (ImageJ v.1.48, National Institutes of Health; available at imagej.nih.gov/ij/download.html). Three measurements of the distance between the skin and the sternum were done for each goat.

Blood samples were taken at the last day of each period from the jugular vein into 10 mL vacutainers with K2-EDTA (BD Diagnostics, Franklin Lakes, NJ) before feeding. Plasma was obtained by the centrifugation of blood for 15 min at 1500 × *g* and stored at –30 °C until the analysis of NEFA, BHB, triglycerides, and cholesterol. The NEFA were determined by colorimetric enzymatic test ACS-ACOD method using a commercial kit (Wako Chemicals, Neuss, Germany). The BHB was determined by kinetic enzymatic method using commercial kit (Ranbut, Randox, UK). Cholesterol was analyzed by the enzymatic method (cholesterol esterase/peroxidase), whereas triglycerides were analyzed with enzymatic method (glycerol phosphate oxidase) using an Olympus analyzer (Olympus AU400, Dusseldorf, Germany). Blood carnitine fractions (free carnitine, acetyl carnitine, and total carnitine) were determined using a quasi-solid phase extraction without derivatization reactions by means of normal-phase liquid chromatography and electro spray ionization tandem mass spectrometry (Applied Biosystems, Darmstadt, Germany) according to Hirche et al. [18].

At the end of each period, additional blood samples (approximately 0.5 mL) were collected by insulin syringes (1 mL; BD Micro-Fine, BD Medical-Diabetes Care, Franklin Lakes, NJ), before feeding and immediately analyzed for major ions and metabolites. A single drop of blood was applied to disposable cartridges containing biochemical and silicon chip technology (i-STAT Chem8+, Abbott Point of Care, Princeton, NJ). Then, the cartridge was inserted into an i-STAT handheld analyzer, and glucose, urea, Cl, Na, K, ionized Ca, total CO_2_ concentration, anion gap, hematocrit, hemoglobin, creatinine, and base excess were obtained.

### 2.3. Statistical Analyses

Data were analyzed by the PROC MIXED for repeated measurements of SAS v. 9.1.3 (SAS Institute Inc., Cary, NC). The statistical mixed model contained the fixed effects of the temperature (TN and HS), dietary supplementation (CON and CAR), experimental day, period; the random effect of the animal; the interactions of temperature × supplementation, temperature × period, supplementation × period; and the residual error. The model considered the possible carryover effects of previous HS periods through the temperature × period interaction. For the data of rectal temperature and respiratory rate measured at 0900, 1200, and 1700, a fixed factor of the hour of day was added to the model. For the data of blood metabolites, and changes of BW and fat thickness, the PROC MIXED was used without repeated measures, and consequently the day effect was removed from the model. Differences between least square means were determined with the PDIFF test of SAS. Significance was declared at *p* < 0.05 and tendency at *p* < 0.10 unless otherwise indicated.

## 3. Results and Discussion

### 3.1. Carnitine Concentrations in Blood

Blood basal concentrations (samples taken before feeding) of L-carnitine fractions are shown in Table 2. All L-carnitine fractions (i.e., free-, acetyl-, and total-carnitine) in plasma increased (*p* < 0.01) almost three times by CAR supplementation in both TN and HS conditions. LaCount et al. [19] reported that carnitine concentrations in plasma and liver increase when rumen-unprotected L-carnitine is administered into either the rumen or abomasum of dairy cows, indicating that both sites of administration are equally effective for increasing carnitine concentrations in tissues. LaCount et al. [13] also reported a linear increase in plasma and milk carnitine concentrations as dietary carnitine concentration increased. Additionally, Carlson et al. [10] supplemented periparturient dairy cows with 0.046, 0.382, and 0.763 g L-carnitine /kg BW^0.75^ in the diet and detected that total carnitine concentration in plasma increased four to 10 times by the two higher doses.

In a preliminary work, we measured the in situ degradability of the CAR product, and observed that 72.8% of CAR dry matter disappeared at 16 h. Despite this high disappearance in the rumen, CAR in the current study was efficiently absorbed, as indicated by the elevated levels of carnitine in plasma (Table 2). This relatively high solubility does not necessarily mean that the L-carnitine was degraded in the rumen. In fact, rumen microorganisms are not able to completely degrade the solubilized L-carnitine, as dietary supplementation with rumen-unprotected carnitine resulted in elevated blood levels of carnitine in dairy cows [10,12,20].

Free L-carnitine represented approximately 75% of total L-carnitine in the plasma of our goats (Table 2). Free L-carnitine concentrations numerically decreased (−14% on average; *p* < 0.13) by HS, and it seems that this free carnitine was transformed to acetyl-carnitine (more than 16% of total carnitine was presented as acetyl-carnitine in the HS group). Similarly, Thomson et al., [21] reported that dairy goats exposed to cold stress experienced decreased blood carnitine levels with lower loss in the milk, which resulted in saving 52 µmoles/d of carnitine that most probably was used for the increment in fatty acid oxidation. Overall, CAR supplemented in the current experiment was absorbed efficiently and was available in blood for metabolism in both TN and HS goats.

### 3.2. Rectal Temperature and Respiratory Rate

The HS goats showed greater (*p* < 0.001) rectal temperatures and respiratory rates than TN goats at 0800, 1200, and 1700 h (Table 3). Compared to TN goats, HSed goats experienced an increment in rectal temperatures (0.65 to 1.25 °C) and respiratory rates (53 to 91 breaths/min), with the highest values recorded at 1700 h when the heat load was at its maximal level. This agrees with the results of Sivakumar et al. [22], Hamzaoui et al. [4], and Contreras-Jodar et al. [6], where goats exposed to HS experience high rectal temperatures and respiratory rates. The increment in respiratory rate under HS conditions is a known mechanism for dissipating heat load by pulmonary evaporation. The supplementation with CAR had no effect on rectal temperature or respiratory rate throughout the day.

### 3.3. Feed Intake and Feed Sorting

Average DM intake decreased in HS animals by 26% throughout the experimental period (1.90 ± 0.10 kg/d vs. 2.58 ± 0.10 kg/d; *p* < 0.001; Table 4). Goats in the current study were in mid lactation and the reduction of DM intake by heat stress was greater than that previously observed in HS dairy goats during late lactation [4]. On the other hand, CAR supplementation did not affect DM intake, which is in accordance with the results found in cows supplemented with 0.046 or 0.382 g L-carnitine /kg BW^0.75^ [10]. However, when cows were fed a high dose of L-carnitine (0.763 g/kg BW^0.75^), DM intake decreased, plausibly because of increased hepatic ATP production [10].

Heat-stressed goats tended (*p* < 0.06) to eat longer particle size compared to TN goats, as the average particle length of their orts decreased by 27% (6.2 ± 0.64 mm vs. 4.5 ± 0.64 mm for TN and HS, respectively; Table 4). Feed sorting against short particles (that contain greater energy content than longer particles) in addition to the fact that feed intake is reduced by HS would exacerbate challenges associated with reduced energy intake. Castro-Costa et al. [23] reported that HS decreases rumen pH in dairy goats eating the same amount of food as TN goats. Although we did not measure rumen pH in the current study, we speculate that our HS goats ate longer particles to manage possible low pH in the rumen. Additional work is needed to explore the relationship between rumen pH and feed sorting behavior under HS conditions. The increased feed sorting for long particles (presumably forage particles) is in contrast to the common nutritional practice of reducing ration forage content during HS [2]. No effect of CAR supplementation on the orts particle size was observed.

### 3.4. Milk Yield and Composition 

As shown in Table 4, milk yield tended (*p* < 0.06) to decrease by 11% in goats exposed to HS (1.63 ± 0.09 kg/d) compared to TN goats (1.84 ± 0.09 kg/d). This reduction was similar to what observed by Hamzaoui et al. [24] in HSed dairy goats at mid lactation. The consequences of high ambient temperature in lactating dairy ruminants are well known [1,2,5], which include increased body temperature, reduced DM intake, and consequently, altered milk yield and composition. Milk composition was also affected by high ambient temperature (Table 4); the HS goats producing milk with lower fat (*p* < 0.08), protein (*p* < 0.05), and lactose (*p* < 0.01) contents than TN goats.

We hypothesized that carnitine synthesis is reduced by the potential inflammation induced by HS, and that CAR supplementation would cover the shortage in the production of endogenous carnitine. Additionally, if the HSed goats mobilize body fat due to the reduced feed intake, supplemented CAR would improve the oxidation of mobilized fatty acids. This could improve the efficiency of energy use and reduce the adverse effect of HS on milk production. However, CAR supplementation did not affect milk yield or milk composition (Table 4), indicating that CAR supplementation has no beneficial effects on milk production of HSed goats. Similarly, LaCount et al. [19] found no effect of CAR supplementation on milk production of non-heat-stressed dairy cows. Nevertheless, feed-restricted dairy cows (with high blood NEFA levels) produced greater 3.5% fat-corrected milk when supplemented with abomasum infused L-carnitine [25]. Furthermore, dairy cows supplemented with rumen-protected L-carnitine, from one week before calving to four weeks after parturition, produced similar milk yield to un-supplemented cows, but their milk contained greater fat and protein [26]. These studies indicate that carnitine supplementation may be required during the situations of DM intake depression that cause body fat mobilization and elevated blood NEFA (e.g., transition period or feed restriction). Although HS in the current study reduced feed intake by 26%, HS and TN goats had similar blood NEFA levels (see the blood metabolites section), which may explain why CAR did not improve the performance of HSed goats.

### 3.5. Body Weight and Subcutaneous Fat Assessment

**Figure 1:**
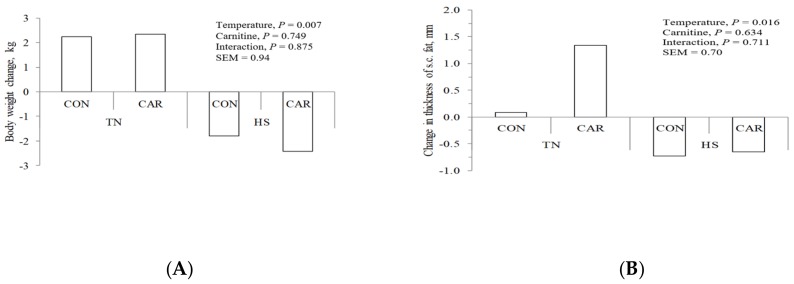
Changes in body weight (A) and subcutaneous fat thickness (B), measured as the difference between values at the start and the end of each experimental period in dairy goats under thermo-neutral (TN) or heat stress (HS) conditions. In each ambient temperature, goats were fed a control diet (CON) or supplemented with 5 g CAR containing 1 g pure L-carnitine.

Changes in BW and subcutaneous (s.c.) fat were expressed as the difference between the values at the start and the end of each experimental period (Figure 1). On average, HSed goats lost 146 g/d of BW, whereas TN goats gained 139 g/d, agreeing with the results of Hamzaoui et al. [4]. A portion of the BW changes of TN and HS goats included the inevitable variations in the digestive tract content (reduced feed intake in HS), which were unknown in our data. Supplementation with L-carnitine did not affect the overall BW variation. This agrees with the results found in growing sheep in which L-carnitine do not affect average daily gain [27].

The values of ultrasound fat measurements (Figure 1) revealed that HSed goats had lower s.c. fat thickness compared to TN (−0.72 vs. +0.64 mm; *p* < 0.05). L-carnitine supplementation did not affect s.c. fat mobilization in accordance with the results of Hajilou et al. [28], who detected no changes in s.c. fat in finishing bulls supplemented with L-carnitine. In addition, BW variation and ultrasound measurements were positively correlated (*r* = 0.54), indicating that HSed goats in negative energy balance would mobilize body fat. Mendizabal et al. [29] indicated that, for the processes of storage and mobilization of fat reserves in adult goats, the s.c. fat was particularly active and appears to be highly specialized for lipid accumulation and mobilization.

### 3.6. Blood Metabolites 

Blood metabolites of TN and HSed goats with and without L-carnitine supplementation are shown in Table 5. L-carnitine supplementation did not affect (*p* > 0.10) blood urea or glucose levels. Previous reports revealed that carnitine supplementation increases insulin secretion and hepatic glucose output by incrementing the flux of metabolites through pyruvate carboxylase [10,30]. In agreement with this notion, Greenwood et al. [20] observed that blood glucose levels increase by 3% in growing steers supplemented with carnitine. Nevertheless, blood glucose in our goats was not affected by CAR supplementation.

Supplementation with L-carnitine did not affect blood NEFA, BHB, cholesterol, or triglycerides concentrations (Table 5). Dairy cows in early lactation have decreased blood NEFA and cholesterol concentrations when supplemented with 10 g/d of protected carnitine [31]. In addition, Citil et al. [32] reported that feed supplementation with L-carnitine decreases serum triglycerides and cholesterol in lactating ewes. Nevertheless, and agreeing with our findings, Carlson et al. [25] reported that plasma NEFA is not altered in mid-lactation dairy cows infused with L-carnitine.

The decrease (*p* < 0.01) in blood urea concentration by HS could be explained by their lower DM intake and, consequently, reduced N intake. Despite the reduced feed intake (Table 4), HSed goats were able to keep similar glucose levels to TN goats. Creatinine levels increased (*p* < 0.01) with HS, which might indicate increased muscle degradation. It is possible that some glucogenic AA produced from muscle degradation were used for gluconeogenesis, resulting in keeping similar blood glucose levels. Additionally, Salama et al. [3] reported that HSed goats secrete lower insulin when glucose is infused compared to TN goats, which might be an adaptation to HS and could explain unaltered glucose levels in HS goats.

Values of total blood CO_2_ were lower in HS compared to TN goats, which agrees with previous studies [4,22]. The greater respiratory rate observed in HSed goats (Table 3) contributed to washing off of CO_2_, and consequently a lower concentration of CO_2_ in blood. The Cl^−^ and K^+^ blood concentrations were greater (*p* < 0.05) in HS than in TN goats. The blood ionized Ca concentration also tended (*p* < 0.10) to be greater in HS (1.29 mmol/L) compared to TN goats (1.26 mmol/L). Similarly, Srikandakumar and Johnson [33] reported greater concentrations of K, Cl, and Ca in blood of heat-stressed cows. Collier et al. [34] reported that K requirements in HSed dairy cows increase by as much as 12% because sweat is high in K. In the current study, blood K in HSed goats increased by 11% to meet K requirements despite the reduced mineral intake. However, our goats had free access to mineral-vitamin blocks, which might have allowed them to obtain minerals as needed.

As indicated above, s.c. fat thickness measured by ultrasonography was decreased by HS (Figure 1), indicating that goats would have mobilized body fat reserves. However, both blood NEFA and BHB did not change by HS in the current study (Table 5) and previous studies done in dairy goats [3] and dairy cows [1]. It is possible that there was some degree of body fat mobilization, but NEFA were rapidly taken up by the mammary gland for fat synthesis (milk fat was less affected by HS than milk protein as shown in Table 4) and were not transformed to ketone bodies by the liver. We detected no correlation between blood NEFA and BHB in TN (r = 0.02; *p* = 0.953) or HS (r = 0.18; *p* = 0.478) goats.

Finally, and from the viewpoint of animal welfare, the level of HS used in the current study was moderate according to the established levels of HS in dairy goats [12]. Furthermore, we used the minimum possible number of goats (n = 4; Latin square four × four) and the shortest time for each period (16 days) to test the effects of L-carnitine. As stated previously, our hypothesis was solid and we expected positive effects of L-carnitine on HS goats, but we were not able to detect such effects. This will avoid unnecessary exposure of animals to HS in the future to test the effects of L-carnitine.

## 4. Conclusions

Heat stress negatively affected the lactational performance of dairy goats. Additionally, heat stress altered feeding behavior as heat-stressed goats tended to consume longer feed particles as an attempt to keep stable rumen pH. Supplementation of thermo-neutral and heat-stressed lactating dairy goats with rumen protected L-carnitine dramatically increased blood carnitine fractions (free-, acetyl-, and total-carnitine). Despite the effective absorption of carnitine, no productive benefits or physiological changes were observed in dairy goats under thermo-neutral or heat stress conditions. Evidence was not obtained to support the hypothesis that carnitine supplementation is needed under heat stress conditions.

## Figures and Tables

**Table 1:** Chemical composition and nutritive value of the ration expressed on dry matter (DM) basis.

Item	Total Mixed Ration
Component, %	
Dry matter	88.2
Organic matter	88.1
Crude protein	17.7
Ether extract	1.79
Neutral detergent fiber	39.3
Acid detergent fiber	28.6
Nutritive value^1^	
UEL,^2^ /kg	1.08
UFL,^3^ /kg	0.76
PDI,^4^ g/kg	94.2
PDIA,^5^ g/kg	45.5
RPB,^6^ g/kg	31.6
Ca_abs_, g/kg	2.73
P_abs_, g/kg	0.84

^1^ Calculated according to the Institut National de la Recherche Agronomique (INRA, 2018). ^2^ Fill units for dairy goats (1 UEL = 1 kg DM of reference grass). ^3^ Net energy for lactation (1 UFL = 1.76 Mcal of NEL). ^4^ Protein digestible in the intestine from dietary and microbial origin. ^5^ Protein digestible in the intestine from dietary origin. ^6^ Rumen protein balance.

**Table 2:** Least squares means for L-carnitine fraction concentrations (µmol/L) in plasma of dairy goats under thermo-neutral (TN) or heat stress (HS) conditions. In each ambient temperature, goats were fed a control diet (CON) or supplemented with 1g L-carnitine (CAR).

L-Carnitine	TN		HS		SEM	Effect ^1^ (*p* =)		
	CON	CAR	CON	CAR		T	C	T × C
Free	20.42	61.23	18.42	50.50	4.06	0.13	0.01	0.30
Acetyl-carnitine	6.79	17.49	6.90	21.24	2.08	0.35	0.01	0.38
Total	26.78	78.30	24.90	71.30	4.32	0.32	0.01	0.56

^1^ Effects of temperature (T), CAR supplementation (C), and their interaction (T × C).

**Table 3:** Least squares means for respiratory rate and body temperature of dairy goats under thermo-neutral (TN) and heat stress (HS) conditions. In each ambient temperature, goats were fed a control diet (CON) or supplemented with 1g L-carnitine (CAR).

Item	TN		HS		SEM	Effect ^1^ (*p* =)		
	CON	CAR	CON	CAR		T	C	T × C
Rectal temperature, °C								
0800 h	38.5	38.5	39.1^c^	39.2^c^	0.05	0.001	0.290	0.146
1200 h	38.6	38.5	39.7^b^	39.7^b^	0.05	0.001	0.520	0.863
1700 h	38.7	38.6	39.9^a^	39.9^a^	0.05	0.001	0.222	0.648
Average	38.6	38.5	39.6	39.6	0.04	0.001	0.762	0.464
Respiratory rate, breaths/min								
0800 h	36	35	88^b^	88^b^	3.0	0.001	0.939	0.933
1200 h	36	36	126^a^	121^a^	3.0	0.001	0.455	0.282
1700 h	40	39	133^a^	127^a^	3.0	0.001	0.247	0.144
Average	37	37	116	112	2.4	0.001	0.430	0.542

^1^ Effects of temperature (T), CAR supplementation (C), and their interaction (T × C). ^a–c^ Values within the same column (at 0800, 1200, and 1700 h) for each parameter with different superscripts differ (*p* < 0.05).

**Table 4:** Least squares means for feed intake, average particle size of the orts, and milk production of dairy goats under thermo-neutral (TN) or heat stress (HS) conditions. In each ambient temperature, goats were fed a control diet (CON) or supplemented with 1g L-carnitine (CAR).

Item	TN		HS		SEM	Effect ^1^ (*p* =)		
	CON	CAR	CON	CAR		T	C	T × C
DM intake, kg/d	2.60	2.56	1.85	1.95	0.16	0.007	0.976	0.859
Orts average particle size, mm	5.31	7.92	3.99	5.02	1.10	0.057	0.238	0.674
Milk yield, kg/d	1.90	1.80	1.59	1.69	0.14	0.059	0.955	0.730
FCM, L/d^2^	2.28	2.15	1.81	1.90	0.19	0.028	0.880	0.765
Milk composition, %								
Total solids	8.89	8.91	8.37	8.46	0.19	0.005	0.926	0.961
Fat	4.33	4.21	4.02	3.96	0.20	0.076	0.729	0.984
Protein	3.51	3.54	3.14	3.22	0.18	0.049	0.951	0.989
Lactose	4.64	4.65	4.47	4.47	0.06	0.006	0.991	0.788
Fat yield, g/d	85.8	80.7	66.5	69.3	7.8	0.015	0.864	0.844
Protein yield, g/d	69.1	64.6	50.3	54.5	6.0	0.008	0.991	0.737
Somatic cell count, Log	5.97	6.00	6.30	6.22	0.23	0.276	0.873	0.842

^1^ Effects of temperature (T), CAR supplementation (C), and their interaction (T × C). ^2^ Fat corrected milk at 3.5%; FCM = kg of milk yield × [0.432 + 0.162 × (fat %)].

**Table 5:** Least squares means for blood metabolites in dairy goats under thermo-neutral (TN) or heat stress (HS) conditions. In each ambient temperature, goats were fed a control diet (CON) or supplemented with 1g L-carnitine (CAR).

Item	TN		HS		SEM	Effect ^1^ (*p* =)		
	CON	CAR	CON	CAR		T	C	T × C
Na, mmol/L	148.0	147.6	147.0	147.0	0.52	0.349	0.881	0.299
K, mmol/L	3.47	3.47	3.84	3.87	0.15	0.042	0.754	0.243
Ionized Ca, mmol/L	1.28	1.27	1.27	1.31	0.02	0.066	0.378	0.310
Cl, mmol/L	103.8	103.6	107.9	107.8	0.67	0.007	0.109	0.497
TCO_2_, mmol/L	25.7	25.9	20.9	21.2	0.69	0.009	0.678	0.871
Anion gap	23.0	22.7	22.8	23.1	0.54	0.707	0.247	0.869
Hematocrit, % PCV	17.6	18.6	17.7	18.4	0.92	0.770	0.609	0.899
Hemoglobin, g/dL	5.98	6.31	6.05	6.26	0.31	0.778	0.599	0.891
Glucose, mg/dL	59.1	59.6	59.9	58.6	1.71	0.743	0.962	0.733
Urea, mg/dL	23.2	24.5	18.3	19.8	1.85	0.008	0.710	0.971
Creatinine, mg/dL	0.47	0.52	0.57	0.54	0.02	0.007	0.906	0.211
Triglycerides, mg/dL	17.6	18.0	17.1	17.1	1.3	0.878	0.660	0.501
Cholesterol, mg/dL	74.7	76.2	85.5	79.9	5.8	0.219	0.911	0.733
non-esterified fatty acids, mmol/L	0.08	0.13	0.15	0.12	0.02	0.291	0.333	0.276
ß-hydroxybutyrate, mmol/L	0.72	0.70	0.80	0.85	0.11	0.187	0.972	0.932

^1^ Effects of temperature (T), CAR supplementation (C), and their interaction (T × C).
